# Brief Notes on the Practical Anatomy of the Shoulder

**Published:** 1898-09

**Authors:** Thomas H. Manley

**Affiliations:** New York; Professor of Surgery, New York School of Clinical Medicine


### BRIEF NOTES ON THE PRACTICAL ANATOMY OF THE SHOULDER.

BY THOMAS H. MANLEY, M. D., NEW YORK.
Professor of Surgery, New York School of Clinical Medicine.

*Mode of Acquiring Special or Practical Anatomy.*—We acquire a knowledge of practical anatomy of a region in various ways:

1. By the aid of treaties on anatomy. This affords us but a relative and indefinite knowledge, because many illustrations are defective, because different authors give different names to the same structure, and, finally, because deviations in development are found in nearly every subject.

2. Dissection on the cadaver—dead anatomy—is the most valuable of all methods in the study of anatomy.

3. The study of the living anatomy is something too much neglected, but of great value. In muscular subjects, devoid of much fat, muscular movements at the shoulder may be studied with advantage.

4. A study of comparative anatomy combined with dissection of the lower animals. No one can pretend to a general knowledge of anatomy who has not given some attention to this side of the subject.

5. By vivisection only can we learn physiological anatomy. This is of inestimable value, if for no other purpose than acquainting ourselves with the mechanism of the parts in motion. Surgical operations are a species of vivisection, and hence, the oftener the surgeon operates the better practical anatomist he becomes.

All of the above is germane to our subject, for everything else considered, the more thorough anatomist the surgeon is, the more valuable his opinion and successful his treatment of shoulder or other lesions will be.

*Physiological Anatomy of the Shoulder.*—The shoulders in man hang suspended from the spine. They afford shelter and protection to the viscera of the upper areas of the thorax; defend the cranium, spine, and chest against injury, and are concerned in prehension.

*Triple Arrangement.*—A striking peculiarity here is the triple arrangement, the vicarious function of various structures, and atypical construction,—three special and definite characteristics.

Thus, there are three bones in the skeleton of the shoulder, three joints, three large superficial muscles, each with three separate heads or origins for the superficial layer; there are three large nerve-cords in the brachial plexus, below the clavicle.

The central segment of the deltoid muscle is rich in fibrous tissue, as is the brachial plexus, and hence both serve as auxiliary ligaments.

*Atypical Arrangement of Structure at the Shoulder.*—All three joints are singularly atypical; the costo-scapular, possessing neither synovial membrane nor ligaments; the humero-scapular, being without independent ligamentous support; and the clavicular scapula, having no muscular investment.

The most powerful, complex in structure, action, and surgical importance of all the shoulder muscles is the *deltoid*, or mantle muscle. All the others acting on the humerus are tributary and subsidiary to it.

Next in importance at the humero-scapular articulation is the triceps, the basal muscle of this joint. The subscapularis ranks a good third, in point of importance in luxations, at this site.

The infra- and supraspinatus, with the teres minor, aid in adjustment and rotation, but are of minor importance. The long head or tendon of the biceps plays an auxiliary part in limiting the advance forward of the humeral articular head; but, like the short head of the same muscle and the coraco-brachialis, its main purpose is either directly or indirectly to maintain a steady traction upward on the humerus.

The pectorialis major occupies an intermediate or somewhat doubtful position as an active factor in humero-scapular dislocations. From the origin, arrangement, and insertion of this muscle, we note that it tends to powerfully draw the shoulder forward; it flexes, rotates, and adducts the arm. It is the great shelter muscle of the axillary hollow.

*Role of Sanguinous Effusion into the Bursas.*—From the experiment of inducing mechanical effusion, it is reasonable to believe that after various types subluxation at the humero-scapular joint such acute bursitis with large effusion may result as to constitute a positive obstacle to reduction, until the inflammatory elements have undergone resorption. Possibly in some instances, during violent efforts to return the displaced head, the distended inflamed bursa may rupture, but this did not occur on the cadaver experimentally.

*The Miss-called Shoulder Joint.—The Humero-Scapular Joint.*—This articulation presents several remarkable features in its anatomical construction.

1. In being an articulation of great strength, and yet having almost no articular surface for the humeral head to play on.

2. While the head of the humerus comes directly in contact with only a narrow, nearly flat, osseous surface, yet above and posteriorly it is well sheltered and powerfully protected by the broad, osseous plate of the acromion process.

3. The capsule is remarkable for its looseness, elasticity, capacity, and toughness. It receives many auxiliaries of dense fibrous structure from the tendon of the subscapularis, the teres minor, and the dense aponeurotic development over the acromion process.

4. So wide a range of action is permitted the humeral head that without the acromio-coracoid arch and the coracoid process the tendency to anterior displacements would be greatly augmented.

5. The articular head of the humerus is maintained in complete position by a single muscle and no accessory aids, as ligaments, atmospheric pressure, or peculiar joint-construction.

*Characteristic Peculiarities at Scapular Extremity of the Clavicle—The Acromio-Clavicular Joint.*—This articulation is an integral component of the shoulder mechanism, though not indispensable to its functional use, and hence, probably, why it is generally so briefly considered by authors on anatomy.

Like the humero-scapular joint, it anatomically is atypical. The head of the clavical plays on a flat osseous surface. A distinct meniscus divides the capsule, and the articulation is entirely ligamentous. This joint derives much support from the dense aponeurotic expansion which extends over the acromoin process and blends with the trapezius and deltoid. Its position is further secured by the subclavian, the pectoralis major, and deltoid muscles. This articulation permits such a range of motion that the whole shoulder may be carried towards or from the central plane of the body without any change occurring in the vertical axis of the humero-scapular joint.

The most remarkable features associated with or which follow dislocation at this articulation are (1) that they are liable to be overlooked, or difficult to detect in fleshy subjects; (2) they are generally irreducible; and (3) if irreducible, they seldon very seriously impair function.

*Faulty Teaching of Anatomy.*—Much has been written about the difficulty of recognizing dislocations at the shoulder; but this controversy can only be settled by an appeal to physiological anatomy; when we must recognize that there is such a state as physiological dislocation, and such a condition as incomplete dislocation, when the articular head of the humerus has separated from the glenoid fossa, partly, but it is yet within the capsule. Strictly speaking, the articular head is somewhat displaced, but there is no true dislocation.

It is time, in the interest of science, that anatomists and surgical teachers explain what they mean by the "head" of the humerus. Which head? The external head or trochanter, being superficial, while the articular or internal, the true head, is everywhere quite impossible to detect by palpation.

These few and many other anatomical points must be now fully and definitely elucidated by anatomists before anything like a precise knowledge of those disorganizations involving the shoulder is possible.

